# Potentiometric pH Sensor Based on Flexible Screen-Printable Polyaniline Composite for Textile-Based Microfluidic Applications

**DOI:** 10.3390/mi13091376

**Published:** 2022-08-23

**Authors:** Yohan Laffitte, Bonnie L. Gray

**Affiliations:** Microinstrumentation Lab, School of Engineering Science, Simon Fraser University, Burnaby, BC V5A 1S6, Canada

**Keywords:** skin pH sensor, wearable pH sensor, textile-based sensor, screen-printable pH sensor, wearable microfluidics, flexible pH sensor, polymer nanocomposite, micro clothing-based analytical systems, screen-printable polyaniline composite

## Abstract

Skin pH can be used for monitoring infections in a healing wound, the onset of dermatitis, and hydration in sports medicine, but many challenges exist in integrating conventional sensing materials into wearable platforms. We present the development of a flexible, textile-based, screen-printed electrode system for biosensing applications, and demonstrate flexible polyaniline (PANI) composite-based potentiometric sensors on a textile substrate for real-time pH measurement. The pH response of the optimized PANI/dodecylbenzene sulfonic acid/screen-printing ink composite is compared to electropolymerized and drop-cast PANI sensors via open circuit potential measurements. High sensitivity was observed for all sensors between pH 3–10, with a composite based on PANI emeraldine base, demonstrating sufficient response time and a linear sensitivity of −27.9 mV/pH. This exceeded prior flexible screen-printed pH sensors in which all parts of the sensor, including the pH sensing material, are screen-printed. Even better sensitivity was observed for a PANI emeraldine salt composite (−42.6 mV/pH), although the response was less linear. Furthermore, the sensor was integrated into a screen-printed microfluidic channel demonstrating sample isolation during measurement for wearable, micro cloth-based analytical devices. This is the first fully screen-printed flexible PANI composite pH sensor demonstrated on a textile substrate that can additionally be integrated with textile-based microfluidic channels.

## 1. Introduction

Advances in wearable technologies have allowed health care providers or consumers to track a multitude of physiological signals, such as heart rate, activity level, and other bio-signals. These sensors can provide real-time monitoring of important health parameters. However, commercially available wearable sensors are typically limited to bioelectric (e.g., heart rate) or physical (e.g., motion) sensing and developed as discrete devices that are then integrated into devices such as watches or patches to provide a housing as well as power and/or computational analysis. Development of textile-based wearable sensors has been limited, despite the potential that such sensors have to be unobtrusively integrated with clothing that a user may wear on a daily basis.

Wearable biochemical sensors have also lagged in development, with only a few blood sampling devices on the market (e.g., glucose monitoring). Alternately to blood, sweat can be collected non-invasively and includes many biomarkers of interest such as glucose, lactate, electrolytes, pH, and urea. Sampling can be conducted passively or actively via chemical or electrochemical stimulation for up to 24 h [1]. Sweat pH has been used as an indicator of dermatitis [2] and related to hydration levels [3]. Equally, skin pH has been used to track infections of healing wounds [4,5].

Many flexible pH sensors have been developed [6,7]. However, these sensors require complex fabrication methods or a clean room environment for fabrication. Screen-printing devices offers a much simpler and cost-effective approach to fabricating sensors and is already used for both electrochemical biosystems and artistic patterning of clothing. For textile-based sensors, current approaches typically involve either printing on clothing (screen-printing) or using functional yarns/threads and then weaving these into textiles. Current limitations include making devices that are simultaneously (1) flexible, (2) adequately functional, and (3) relatively simple to manufacture. A benefit of textile-based sensors relates to user comfort, acceptance, and compliance [8]; the ability to integrate wearable devices directly into clothing has the possibility of improving user compliance and acceptance by improving wearer comfort. Clothing is already a normal part of people’s daily routines, so including devices that can monitor physiological responses without changing people’s lifestyles could provide a greater benefit to long-term monitoring applications. Other competing technologies include skin tattoos or stickers that can be applied to the skin directly [9]. Previously, our research group has integrated screen-printed polymer nanocomposite electrodes for fluid impedance sensing with microfluidics on textiles [10]. However, while demonstrating the fabrication technology, it did not measure a physiologically relevant analyte such as pH.

Flexible carbon-based pH sensors have been developed by others via screen printing [11], but ultimately suffer from low sensitivity (−11 mV/pH). Comparatively, polyaniline (PANI) has shown sensitivities as high as −60 mV/pH [9,12,13,14,15]. PANI is a polymer whose conductivity can be reversibly modified through protonation, providing an ideal transduction mechanism for pH sensing. It is also biocompatible and environmentally stable. However, PANI processing still presents a challenge, as its conductive emeraldine-salt (ES) form is insoluble in most solvents. Moreover, most PANI sensors reported in literature are electropolymerized in situ. Although this method is reliable and produces highly sensitive sensors, it is not a manufacturing process that is as readily scalable as printing processes, where all parts of a sensor are printed using the same methods.

PANI powders can also be easily synthesized through chemical oxidative polymerization of aniline [16]. The deprotonated non-conductive form of PANI, emeraldine base (EB), is then somewhat soluble in aprotic polar solvents. It can be made even more solution-processable via the addition of organic acids such as dodecylbenzenesulfonic acid (DBSA) and camphorsulfonic acid (CSA). These components have the dual purpose of acting as plasticizers and dopants, imparting a linear conductivity response between pH 3–9 [17,18]. Some of these advancements have led to inkjet-printed and drop-casted PANI using CSA [18] or combined with multi-walled carbon nanotubes (MWCNTs) [19] and graphene [20]. For example, Gill et al. created a screen printable PANI composite, which they printed onto a rigid alumina substrate to form a resistive pH sensor [21].

Despite PANI’s advantages as a pH-sensing material, no pH sensors have been previously developed in which the entire sensor, including a PANI-based working electrode (WE), have been fully screen-printed on a flexible, much less a textile, substrate. Furthermore, flexible pH sensors, including screen-printed ones, are typically designed and tested as stand-alone devices, without being integrated with equally flexible microfluidic systems. Such integration enables the isolation of sensor and assay components from external contamination to ensure reliable sample collection, protects the sensor surface from mechanical abrasion, and prevents sample alteration due to evaporation.

Our group has previously demonstrated screen-printable silver nanoparticle composites for biopotential monitoring [22]. Building on this work, we have also presented initial results towards flexible, screen-printable PANI composite inks for textile-based, wearable potentiometric pH sensors [23]. This work is significant, as, to the authors’ knowledge, the first fully screen-printed flexible PANI composite pH sensor demonstrated on a textile substrate. We have also previously presented 3D cloth-based microfluidics with an embedded pH sensor [24] towards wearable micro clothing based analytical systems (µCAD). The current work combines and expands results from these latter two conference publications, including additional flexibility, fabrication, and sensor response data. This gives a more complete picture of the sensor’s simplicity of fabrication, flexibility as a wearable device, and potential ease of integration with wearable microfluidic systems, where the entire system may be fabricated using the same simple screen-printing process. Moreover, the sensitivity of this screen-printable composite sensor is greater than previous fully screen-printed pH sensors, which holds even when the sensor is flexed, as may be seen when integrated with clothing and worn. This work thus demonstrates the operation and flexibility of the fully screen-printed electrochemical pH sensor comprised of a screen-printed PANI composite as the working electrode on a textile substrate, without any further processing.

## 2. Materials and Methods

In this section, the materials and fabrication processes for the textile-based pH sensors with three different types of working electrodes (WEs) are presented: drop-cast, electropolymerized, and composite polymer. While our focus is primarily on fully screen-printable sensors with composite polymer WEs, we include the other two types as alternate working sensors (albeit with somewhat more complicated fabrication processes), and for comparison. In addition, we present the materials and fabrication methods for integrating the composite polymer-based sensors with microfluidic channels in a textile substrate, and the experimental equipment and methods used to test the pH sensors’ responses and flexibility.

### 2.1. Materials

Aniline, DBSA (95%), PANI-EB (M.W. ~10,000), PANI-ES (M.W. ~20,000), standard buffer solutions from pH 3–10, potassium ferricyanide, HCl, KCl, heptane, and carbon/graphite screen-printing paste were all obtained from Sigma-Aldrich. Plastisols (P-5011 curable reducer and Unistretch 9160) were ordered from The Screen Print Store (thescreenprintstore.ca) and water-based Speedball^®^ White Screen-Printing Ink was bought from Opus Art Supplies. AG-500A silver conductive ink was obtained from Kayuka Advanced Materials, and AgCl (113-09) ink was purchased from Creative Materials to create screen-printable conductive traces and reference electrodes (RE), respectively. Clear Self-Adhesive Protective Book Cover Tape, used as a mask in lieu of a screen for screen-printing, was acquired from Staples Business Depot. A 100% polyester textile substrate was purchased from Fabricana and employed as the substrate for all devices. All materials were employed as purchased.

### 2.2. Fabrication

**Figure 1:**
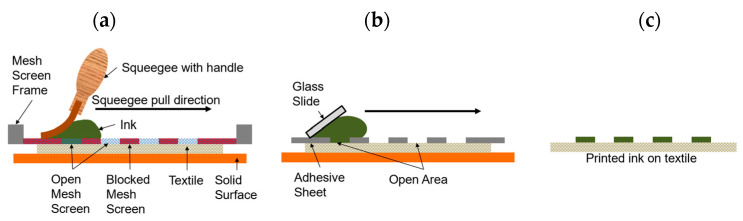
(**a**) Graphical representation of typical textile screen-printing; (**b**) graphical representation of screen-printing process used in this work, which replaces the mesh screen with a laser cut adhesive sheet; and (**c**) finished product of both processes.

Screen printing is a widely used patterning technique for various substrates, including textiles. Essentially, ink is poured onto a patterned mesh screen which sits in proximity to the substrate. The viscosity and surface tension of the ink prevents it from leaking through the screen. Then to pattern the substrate, the screen is brought into contact with it and a blade or squeegee is pressed against the screen to squeeze the ink through the mesh holes and onto the fabric (Figure 1a). The ink adheres to the substrate as the screen is removed, and the substrate is flash heated to quickly dry and set the ink. For the work presented here, the screen-printing follows this conventional process, except with an adhesive layer stencil instead of a mesh screen (Figure 1b). Stencil printing and screen-printing are often used interchangeably in literature, with stencil printing being performed more readily, as it is simpler for rapid prototyping.

The most popular inks used in the textile screen-printing industry at this time are plastisol-based. These inks are made up of plasticized PVC microparticles, contain no solvent, and cure upon heating. The second most popular screen-printing inks are water-based inks with PMMA particles, such as Speedball^®^, which are more environmentally friendly given their absence of PVC. Both inks are well-characterized and already optimized for screen-printing on textiles.

#### 2.2.1. Screen-Printed Electrodes and Design Validation

All screen-printed electrodes (SPEs) on textile substrates were prepared using the same procedure, with only the WE preparation and materials being different for the three different sensors: drop-cast WE, electropolymerized WE, and composite polymer WE. All patterns were created in Corel Draw and transferred to an adhesive sheet using a Universal Laser Systems CO_2_ laser cutter operating with optimized parameters at 13% power and 100% speed using a vector scan. The patterned adhesive sheets were then used as a stencil for printing of various inks onto the textile substrate.

**Figure 2:**
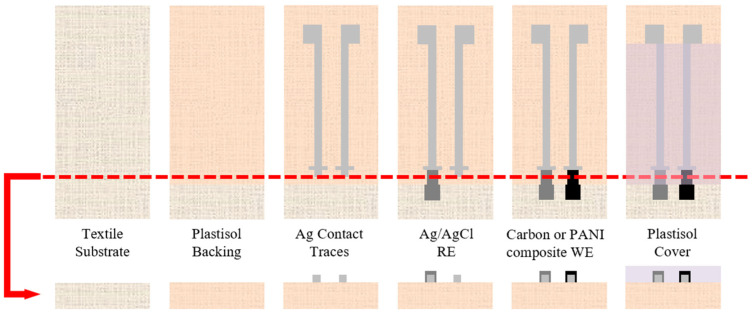
Fabrication process for textile-based SPEs showing top view (**top row**) and cross-sectional view (**bottom row**); all layers are screen-printed sequentially in order from left to right.

Figure 2 illustrates this fabrication process and the order of the printed layers. Initially, a portion of the polyester textile substrate is coated with P-5011 plastisol to create an impermeable backing. Next, silver ink is deposited to create contact pads and conductive traces to the electrodes along with alignment marks for the electrodes. Ag/AgCl ink is then printed to create the RE and either the carbon/graphite conductive paste or the PANI composite are used to create the WE. Lastly, Unistretch 9160 is printed over a portion of the electrode and conductive traces to isolate the conductive traces from each other while in solution.

**Figure 3:**
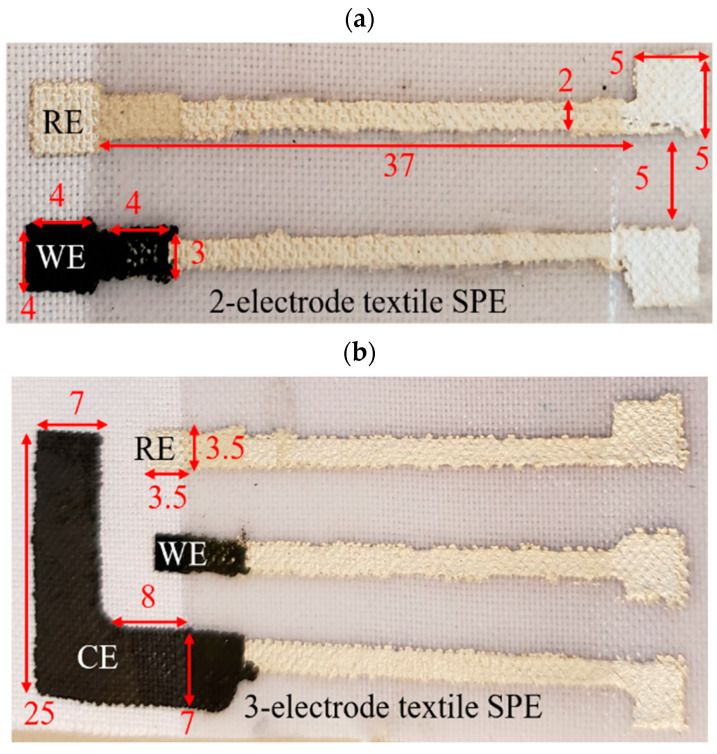
SPE sensor designs showing lateral dimensions in millimeters: (**a**) 2-electrode design; (**b**) 3-electrode design (**a**) adapted from [23]).

Example dimensions for the SPEs for both two-electrode and three-electrode sensors are given in Figure 3. These dimensions were selected to be comparable to commercial SPEs from Pine Research, which were used to validate the design. Additionally, these dimensions were selected so that the sensor could be submerged in solution and still be connected to the test equipment. The device could be further miniaturized, but given the manual fabrication followed in this work, these dimensions were selected to achieve consistent reliable prints by the author.

When a three-electrode system was required, a counter electrode (CE) made from carbon paste was printed next to the WE, as shown in Figure 3b. The CE area was at least 20 times that of the WE to ensure the measured current was that of the WE [25]. The different layers were achieved using adhesive sheets with different patterns cut into them. After the ink was printed onto the textile, the patterned adhesive sheet was carefully peeled off to not disturb the recently printed ink layer. The inks were then cured on a hotplate according to their manufacturer specifications at 130 °C. The PANI composites were cured for 60 s. Once cured, these ink layers do not peel off after a tape test, allowing additional layers to be patterned overtop using this approach. The tape test consisted of applying a layer of Scotch Magic Tape (810) to the printed test line by hand and peeling it off. A discontinuity of the electrical pathway after a tape test was considered a failed tape test. Failed tests also showed visible residue on the adhesive tape surface.

**Figure 4:**
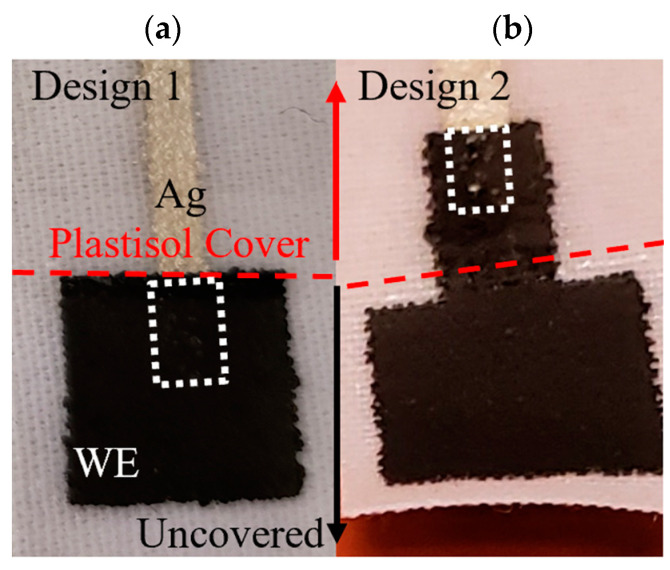
(**a**) Design 1 and (**b**) Design 2, considered for the connection between the electrode and the conductive trace of the textile SPEs. The red dashed line indicates how far the plastisol cover extend down; the white trace indicates how far the Ag trace extends under the WE.

One main design consideration for the textile SPEs shown in Figure 4 was the connection between the conductive traces and the electrode material. Two designs, shown in Figure 4, were initially considered. The first had a conductive trace that extends beyond the plastisol cover and under the WE ink, whereas the second design had the WE material extending under the plastisol cover. As discussed in Section 3.1, Design 2 was deemed superior due to the porosity of the WE material.

#### 2.2.2. Electropolymerized and Drop-Cast Sensors

Sensors with either an electropolymerized or drop-cast WE were developed with a carbon/graphite paste WE, as described in Section 2.2.1. The WE was then modified by either electropolymerizing or drop-casting PANI onto it. The electropolymerized PANI was prepared by first mixing aniline into a solution of 1 M HCl for 1 h with a magnetic stir bar. The reaction was then carried out by cycling the potential between 1.0 V and −0.2 V for 20 cycles at a rate of 40 mV/s at room temperature. The drop-cast sensors’ WEs were prepared by drop-casting a solution of 5 mg/mL PANI-EB in heptane with 1 molar equivalence of DBSA. These solutions were then sonicated for 10 min and filtered with a 0.2 µm syringe filter to remove any large, unsuspended particles. A few droplets (approximately ~50 µL) were then pipetted to cover the carbon paste WE using a plastic pipette and allowed to dry in air. The formation of these films was confirmed by the characteristic CV curve of PANI using a three-electrode textile SPE sensor.

#### 2.2.3. Screen-Printable PANI Composite Ink Sensors

Screen-printable PANI composite inks were prepared by thoroughly mixing PANI-EB or PANI-ES with DBSA and Speedball water-based textile screen-printing ink, using a metal spatula. Several droplets of water were added dropwise to the composite ink to modify its viscosity and assist with the mixing of the ink components. Various formulations of screen-printing ink were prepared and printed to determine the optimal ink formulation. The optimal ink formulation was determined based on a combination of sensor response, print quality, and flexibility, whose results are described in Section 3.

#### 2.2.4. Enclosed Microfluidic Channels, µCADs, and Sensor Integration

**Figure 5:**
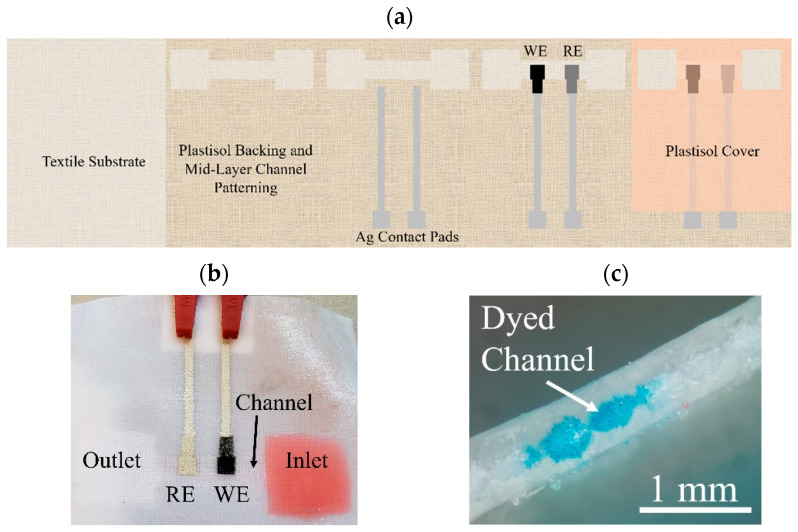
(**a**) Fabrication of embedded SPE in microfluidic channel (**b**) finished device (**c**) textile microfluidic channel cross-section. The smallest printable channel was approximately 1 mm in width (reproduced with permission from [24]). The depth of the channel was approximately 300 µm.

Enclosed microfluidic channels were fabricated within the textile substrate by using a combination of plastisol inks of different viscosities. The low viscosity P5011 plastisol saturated the textile where it was printed, whereas the highly viscous Unistretch 9160 sat atop the textile substrate surface with minimal permeation into the textile layer. In this fashion, an enclosed microfluidic channel was fabricated by outlining the channel with P5011, and then adding a top and bottom layer of Unistretch 9160. The PANI composite ink could then be printed at the interface of this channel by covering the electrodes with the Unistretch 9160 plastisol, resulting in an integrated microfluidic channel/pH sensor device. This device is shown in Figure 5. The hydrophilic textile channel creates fluid flow by wicking fluid, which interacts with the sensor surface. The channel is shown in Figure 5 with dyed solutions. Channel thickness can be controlled based on the textile selection, and the viscosity of the hydrophobic, screen-printed plastisol ink.

### 2.3. Experimental Methods

A Parstat 4000 potentiostat was used with VersaStudio software for PANI synthesis via electropolymerization, as well as for screen-printed electrode and sensor characterization via open circuit potential (OCP) and cyclic voltammetry (CV). A standard Ag/AgCl reference electrode (RE) was used as purchased from Cole Parmer in the 2-electrode SPE sensor designs. The pH of the purchased buffer solutions was verified with an Apera PH700 benchtop digital pH meter. Buffer solutions with pH values from 3 to 10 to cover the range of physiologically relevant pH levels [4,26]. In addition, KCl electrolyte was added to each buffer solution to a concentration of 50 mM to simulate human sweat concentrations. To measure the pH response, the textile SPEs were submerged in a 25-mL beaker of standard buffer solutions with an added 50 mM KCl electrolyte to simulate concentrations in human sweat. The OCP was allowed to stabilize before swapping the pH of the test solution. Sensors were generally determined to be stable when the change in potential was below 0.6 mV per minute, which roughly corresponded to the measured drift rate. The drift level was measured by placing the SPEs in pH 6 buffer solution and measuring the OCP under static conditions.

Resistance measurements for determination of composite polymers’ percolation thresholds were performed with an Agilent U1272A Handheld Digital Multimeter. Percolation threshold is the critical concentration where a composite changes abruptly from non-conducting to conducting as conductive pathways are formed [27]. Thickness measurements of the printed layers were performed using a Tresna Digital Micrometer. Specifically, the textile thickness is measured, then it is measured again when saturated with plastisol, and that measurement is subtracted from the first. The thickness is then measured after each new print is added to determine each layer’s thickness.

**Figure 6:**
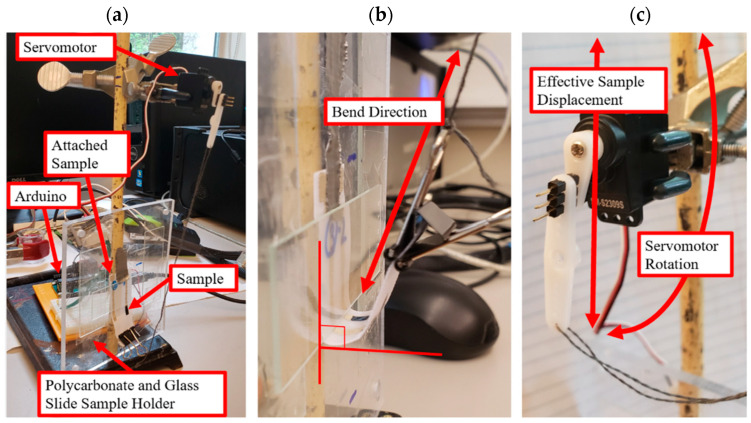
(**a**) Apparatus for effecting multiple bend cycles; (**b**) sample attached to servomotor with binder clip bent to 90° angle; and (**c**) servomotor programmed via Arduino to bend sample.

Bend cycle tests were performed to get a more representative measurement by simulating the bending that may occur to the device when being worn, where multiple deformations are likely to occur. Bend cycles were performed using a custom-built sample holder and servomotor controlled by an Arduino (Figure 6). The sample to be tested was fixed to a plexiglass plate by taping one end of the substrate to the plexiglass. An assembly of three glass slides was fashioned over top of the sample to restrict the bending to the area of interest on the sensor. Two glass slides acted as spacers, with a third to force the sample to bend upon rotation of the servomotor. The unfixed end of the sensor substrate was then attached to the servomotor located directly above it with a binder clip and taut string. An Arduino microcontroller was then programmed to rotate the servomotor position by 180° and back with 1 s breaks in between for a fixed number of cycles. This motion generated a 90° bend in the sensor.

## 3. Results

### 3.1. Screen-Printed Electrodes Design Comparison

**Figure 7:**
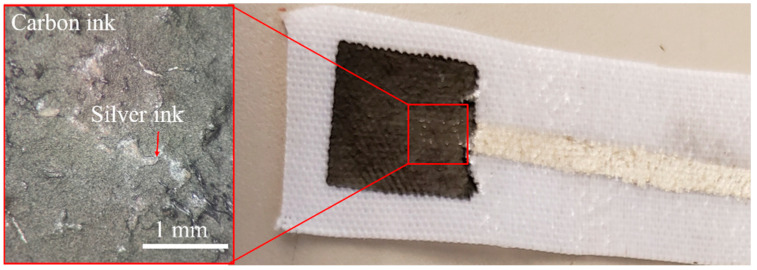
Textile SPE design with silver conductive trace extending past plastisol cover and under the carbon trace and close-up of exposed silver ink under carbon ink.

**Figure 8:**
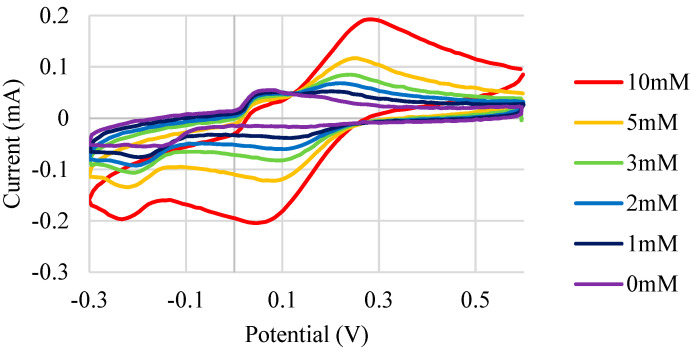
CV curves with K_3_Fe(CN)_6_ as a redox mediator for Design 2.

In theory, both Design 1 and Design 2 shown in Figure 4 should behave similarly. However, it was determined that the WE material was slightly porous after printing. As a result, the Ag conductive trace could be seen breaching the surface of the WE, which affected the measurements of the textile SPE (Figure 7). The CV curves expected using K_3_Fe(CN)_6_ as a redox probe were thus only seen for Design 2 (Figure 8). Design 2 was thus selected, as Design 1 did not show the characteristic peak location for ferricyanide. It also did not show a reversible response (peak in one location). Design 1 could likely still be used if the print had not been slightly porous, or if the printing was carried out in a more automated fashion and not manually, as for this proof-of-concept study. Thus, Design 2 was employed for all subsequent sensor development.

### 3.2. Screen-Printed PANI Composite

The optimization of the screen-printed PANI composites was determined based on the sensor response, the print quality, and the conductivity of the PANI. Initial assessments of the PANI composites were performed by printing test lines and measuring their resistivity. To test the print quality and conductivity of all the various formulations prepared during the composite optimization, 14 test lines, each with a length of 1 cm and a width of 1 mm, were printed for statistical purposes. Various composite formulations for different wt% PANI-EB + DBSA + Speedball were assessed; their respective compositions and average thickness (cross-sectional depth) are given in Table 1. Print thicknesses were mainly employed to calculate resistivity.

**Figure 9:**
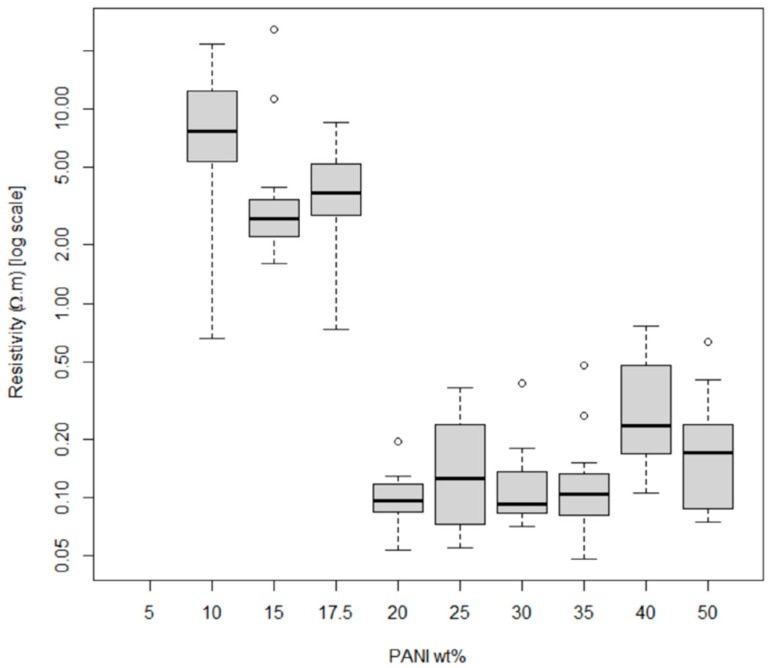
Average resistivity of 14 test lines printed at each wt% of PANI-EB + DBSA + Speedball composites shown in Table 1 (reproduced with permission from [23]). Circles show outlier data that fall outside the quartiles generated by the box plots using statistical software *R*.

The resistivity results of these various formulations are shown in Figure 9. As the concentration of PANI is increased above 10 wt% PANI in the composite, the resistivity generally decreases, as expected. Above 20 wt% PANI, a sharp decrease in the resistivity was observed, indicating the percolation threshold had been reached. Above 35 wt% PANI, the resistivity increases again as the print quality starts to degrade and the prints could be seen cracking and not adhering well to the textile substrate. Adhesion of the films to the substrate was also very poor, where adhesion was deemed adequate when a tape test could be repeated 10 times.

**Figure 10:**
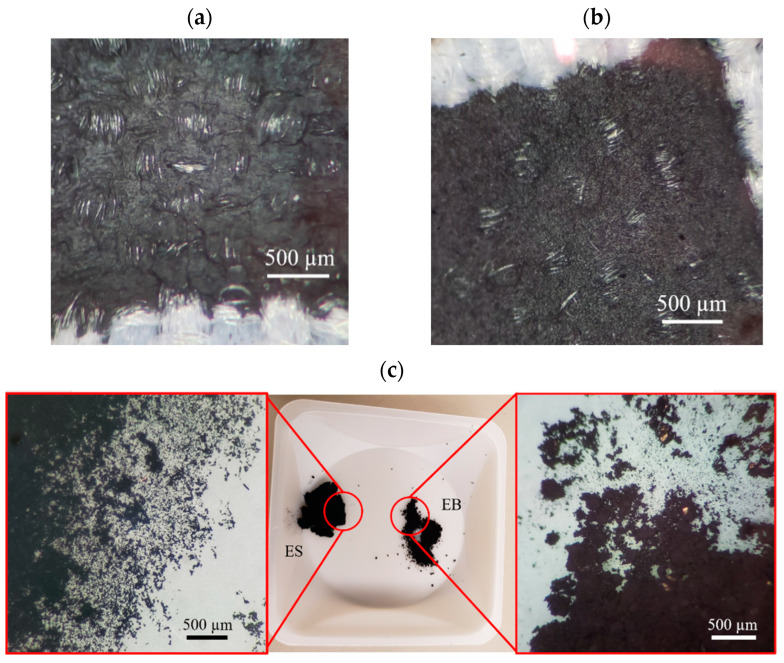
(**a**) PANI-EB + DBSA + Speedball and (**b**) PANI-ES + DBSA + Speedball composites screen-printed on textile substrate showing printing quality. (**c**) Comparison of PANI-ES and PANI-EB particles aggregates, showing much larger PANI-EB aggregates.

Thus, a trade-off exists between qualitative print quality and lower resistivity for the PANI composites at higher (>30 wt%) PANI. Composites with PANI at lower wt% (30 wt% and lower) exhibit similar qualitative print quality regardless of resistivity. The optimal PANI formulation was selected to be 30% PANI for both PANI-EB and PANI-ES formulations to obtain an adequate balance of conductivity and print quality. Low resistivity values were more ideal for use in an SPE and given that the lowest resistivity was seen at 30% weight, this was selected as the optimal formulation for all composite variations. With the optimal PANI-EB formulation selected, a comparison was made between the PANI-EB and PANI-ES composites. Despite the PANI-EB having a smaller molecular weight (~10,000 g/mol) compared to the PANI-ES (~15,000 g/mol), the PANI-EB formed larger aggregates which did not incorporate as well into the composite ink. This can be seen in Figure 10, where the ES particles were on the sub-micrometer scale, whereas some of the EB particles were on the order of a hundred micrometers. As a result, the PANI-EB composites had a much bulkier appearance when printed with more cracking observed compared to the more uniform PANI-ES composite prints (see Figure 10).

A full percolation threshold for the PANI-ES was not performed. It was found that the PANI-ES composites could technically be stencil printed with little to no Speedball screen-printing ink, but these had a tacky, uncured texture and an unreliable pH response. Thus, the optimal PANI-ES formulation was deemed to be roughly similar to that of the PANI-EB composites. The optimal PANI-ES composites withstood 10 sequential tape tests without residue and had resistivities on the order of 10^−3^ Ω-m.

### 3.3. Sensors pH Responses

To fully evaluate the response and sensitivity of the SPE sensors with PANI composite WEs, similar SPE sensors with electropolymerized and drop-cast WEs were also developed and tested for comparison. It should be noted that all three types of sensors were successful and could be employed for pH measurements in the same physiologically relevant range of pH 3–10; however, only the sensors with composite polymer WEs are fully screen-printable and easily integrated with textile-based microfluidic channels for flexible systems.

#### 3.3.1. RE Drift

**Figure 11:**
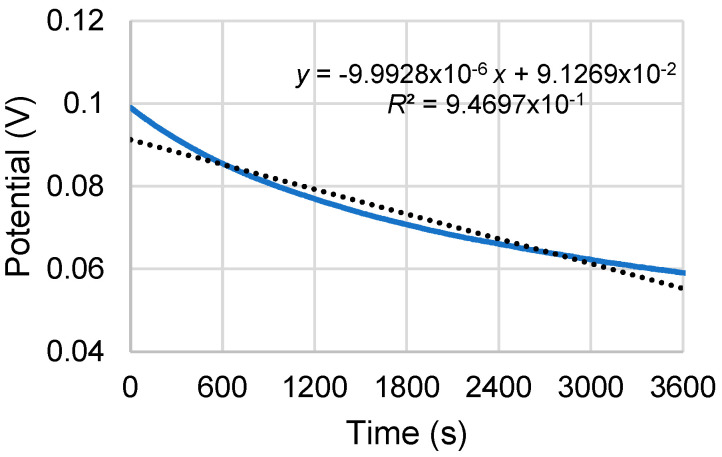
Drift for the Ag/AgCl RE.

The RE was determined to drift as can be seen in Figure 11 for a textile SPE in a pH 6 solution. The slope of the drift was approximately 0.01 mV/s (or 0.6 mV/min) and was used to determine when the sensor had reached steady state after a pH change.

#### 3.3.2. Screen-Printed PANI Composite Sensors

**Figure 12:**
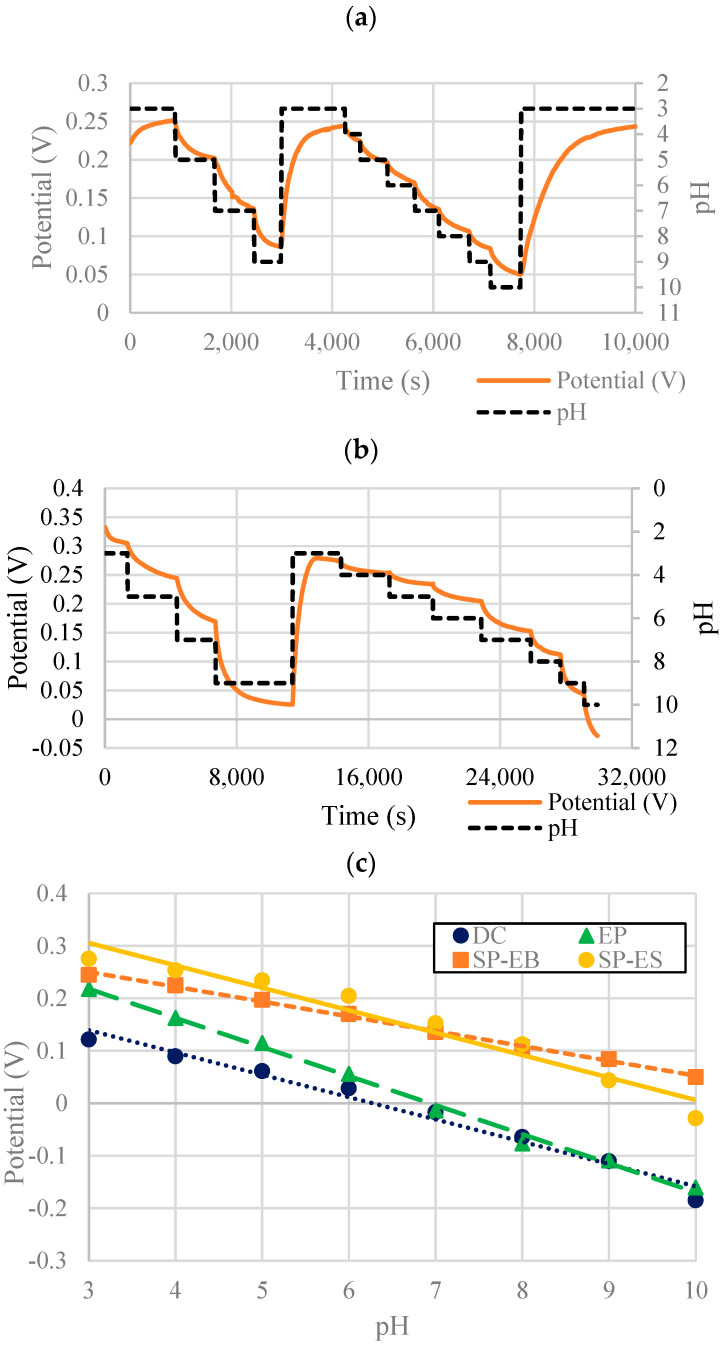
(**a**) PANI-EB + DBSA + Speedball (30/30/40) optimal formulation OCP pH response; (**b**) PANI-ES composite optimal formulation OCP pH response; (**c**) sensitivity of screen-printed composites with PANI-EB (SP-EB) and PANI-ES (SP-ES) with comparison to sensor sensitivities with electropolymerized (EP) and drop-cast (DC) WEs. ((**c**) adapted with permission from [23]).

Figure 12 and Table 2 show the OCP responses and sensitivities of the optimized PANI-EB and PANI-ES composites.

As shown in Figure 12c and Table 2, the PANI-EB composite had a very linear response, with a sensitivity of −27.9 mV/pH. Also shown for comparison are the sensitivity graphs and data for sensors with drop-cast (DC) and electropolymerized (EP) WEs (−41.3 mV/pH and −55.4 mV/pH, respectively). The PANI-ES composite had a sensitivity of −42.6 mV/pH, which is higher than PANI-EB and on par with sensors with drop-casted WEs, but the response was somewhat non-linear. Both PANI-EB and PANI-ES showed fully reversible responses when accounting for the drift.

### 3.4. Sensor Flexibility and Effect on pH Response

**Figure 13:**
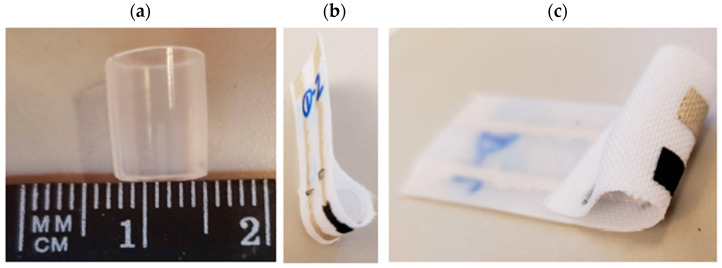
(**a**) Cross-section of disposable 5 mL plastic pipette used for setting radius of curvature of textile SPEs for flexibility testing; (**b**,**c**) Bent textile SPE with PANI composite WE and screen-printed Ag/AgCl RE.

To test flexibility, a sensor with a WE of PANI-EB composite was wrapped around a surface with a radius of curvature of 4 mm (micro-pipette cross-section) and stapled to itself, as seen in Figure 13. We see that the sensor is qualitatively very flexible; however, we note that unlike some other flexible pH sensors, it is not stretchable.

**Figure 14:**
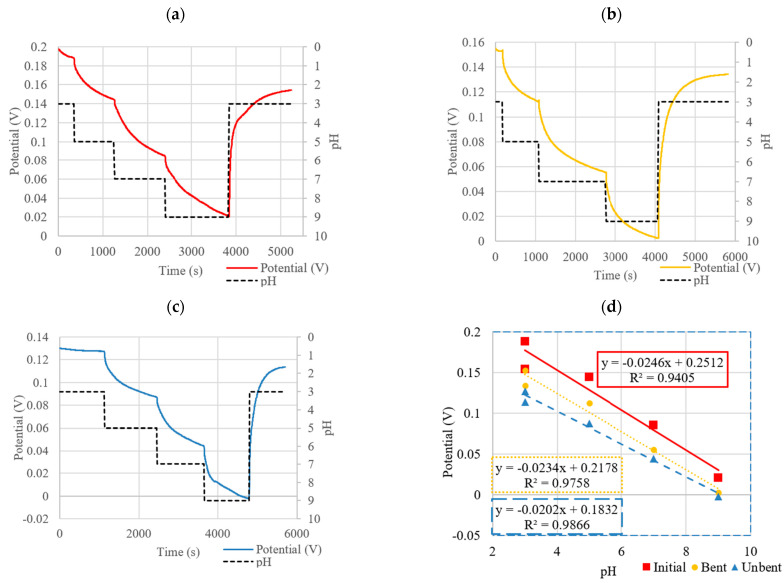
OCP of PANI-EB composite in (**a**) initial state, (**b**) bent state, and (**c**) unbent state along with (**d**) corresponding sensitivity curves.

**Figure 15:**
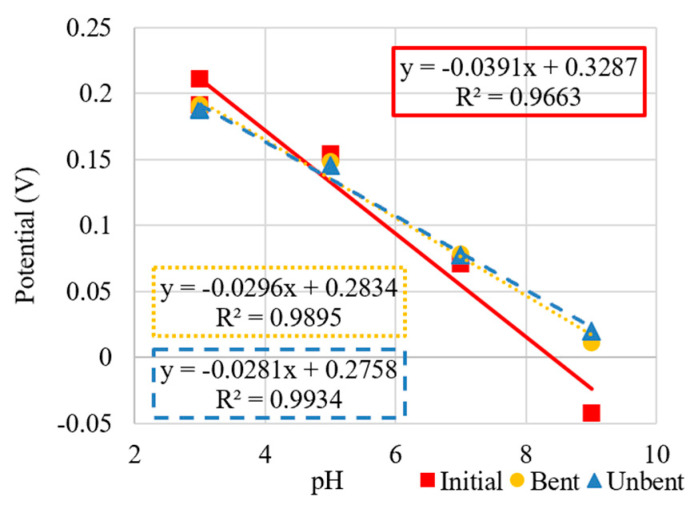
Sensitivity curves of PANI-ES composite in initial, bent, and unbent states.

The OCP was then recorded for the sensor before bending, in a highly bent state, and then again after unbending. The results of these bending experiments are shown in Figure 14 and Figure 15 both for the PANI-EB and PANI-ES composites, respectively. The measurements for the sensor in each case (initial, bent, after unbending) were allowed to continue for different time intervals until each measurement was considered to reach steady state (as described in Section 2.3). For PANI-EB, the sensitivities remained nearly identical. However, a potential drift was seen as indicated by the shifting of potential at comparable pH values, even during the same scan as indicated by the recovery level. The PANI-EB composites showed a slight loss in sensitivity after bending and unbending when compared to the initial response. Additionally, a relatively strong potential drift was observed over the course of these set of responses. This can be seen from the difference between the start and end potentials of the same scan or from scan to scan.

The PANI-ES composites initially showed higher sensitivity but also higher non-linearity, with increasing potential differences as the pH was decreased (see Figure 15). After bending the sensor to the extreme radius of curvature of 4 mm, the sensitivity dropped to levels of the PANI-EB sensors but held this value after unbending, as seen Figure 15.

**Figure 16:**
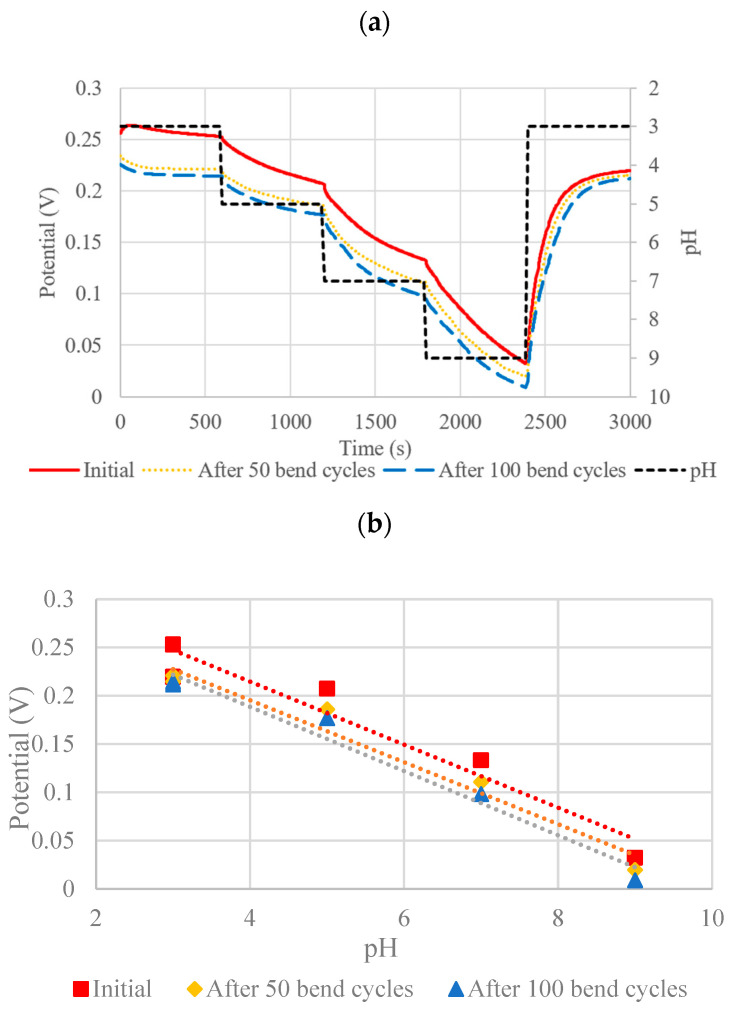
(**a**) OCP pH Response of PANI-ES + DBSA + Speedball composite after 50 and 100 bend cycles and (**b**) corresponding sensitivity curves.

To further quantify flexibility, the PANI-ES sensors were subjected to 90° bends for up to 100 bend cycles using a custom test setup with an Arduino and stereomotor, as described in Section 2.3. Figure 16 shows the OCP results of the sensor after 0, 50, and 100 bend cycles. Although a slight drift can be observed in the potential as indicated by the intercept of the sensitivity curves as seen in Figure 16 and Table 3, the slopes remain relatively unchanged, thus demonstrating good flexibility and robust response under cyclical bending of the sensors with PANI composite WEs.

### 3.5. Embedded Microfluidic Channels and µCADs

To isolate the sensor area from external contaminants, contact with the wearer, and other physical abrasions and prevent evaporation, which could affect measurements, enclosed textile-based microfluidic channels were created to house the composite sensors. Within such a channel, the screen-printed PANI composite pH sensor could then be printed. We have previously shown the flexibility of these channels by operating a textile-based microfluidic device (Y-shaped mixer) in a bent state [24].

**Figure 17:**
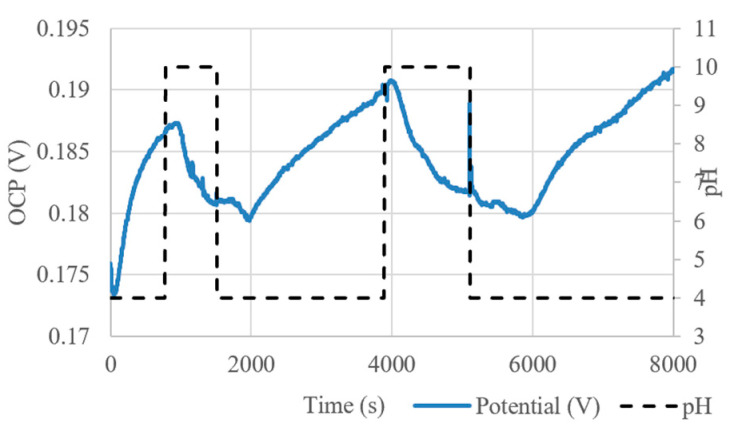
OCP response of embedded SPE (reproduced with permission from [23]).

These channels were used to enclose a fully screen-printed pH sensor with PANI composite WE. The embedded microfluidic channel is 1-mm-wide and 285-µm-thick, and integrated with a miniature, flexible pH sensor with an expected sensitivity of −28 mV/pH. As can be seen from Figure 17, these initial results show a response with a significant time delay and reduced pH response, likely due to the slow filling of the channel by capillary action. However, the integrated microfluidic channel and fully screen-printed sensor does show a real-time response to varying pH levels.

## 4. Discussion

In this work, flexible pH sensors made entirely from easily patterned screen-printable materials, including a novel PANI composite for the WE, were shown to have better sensitivity compared to other fully screen-printed sensors. The sensors also demonstrated a linear response and simple fabrication compatible with textile-based microfluidic channels but suffered from a slow response. Table 4 compares the fabrication methods, flexibilities, sensitivities, and response times (when shifting from solutions with pH 9 to pH 3) of the various sensors on textile substrates in this work and adds those of other closely related work from the literature (fabricated on both textile and non-textile substrates; flexible and non-flexible) for comparison. Evaluating their response to that of the electropolymerized or drop-cast PANI on the textile SPEs, the PANI composite polymer sensors display a slightly lower sensitivity and longer response time. While PANI-ES sensors show a higher sensitivity (−42.6 mV/pH) it had a slower response time of PANI-EB and the response is more non-linear, although this may be acceptable depending on the required pH range of measurement for a given application. It is expected that a higher sensitivity is obtained for the electropolymerized and drop-cast sensors, as these are created using networks of pure PANI. In comparison, the composite networks are interrupted by the supporting polymer matrix, reducing the sensor sensitivity and response time. It should be noted that the sensors described herein achieve 90% of their steady state OCP response in less than half the listed response times of Table 4, notably: 482 s, 838 s, 221 s, and 69 s for the SP-EB, SP-ES, EP, and DC sensors, respectively.

Despite these differences, the advantage of fabricating a sensitive pH sensor using only screen-printing processes represents a significant benefit in terms of scalability and ease of production of devices. In addition, the ability to integrate these sensors into enclosed microfluidic channels within a µCAD also using a fully screen-printable process gives the overall device additional robustness and reliability in reducing sources of interfering contamination. Utilizing the wicking nature of textile materials allows for the continuous exchange of solution and thus operation of the sensor device. Printing sensors onto other flexible substrates such as polyimide are valid alternatives but would still necessitate integration onto a device or substrate (ex: a textile) that exchanged fluid in a reliable manner.

Furthermore, the screen-printed PANI composites demonstrate sensitivities that are above any other fully screen-printed flexible pH sensor [11]. When considering that the expected sensitivity according to the Nernst equation should be −59.1 mV/pH, the results for all three types (EP, DC, SP) of sensors show promise.

Numerous other research groups have developed electropolymerized PANI-based flexible pH sensors on various electrode substrates. These have included bandages [13], tattoos [9], and nano-patterned electrodes [14] which have all achieved near-Nernstian responses with time responses on the order of several to tens of seconds. These sensitivities are in line with those reported in this paper for electropolymerized PANI pH sensors; however, the response times were quicker than those observed here. Despite the promising results in these prior publications, these methods lack the simplicity in fabrication that can be achieved from a fully printed approach.

Gill et al. [21] developed a PANI-PVB-PS3 composite that could be screen-printed on rigid substrates for pH sensing applications. Although effective, it was not shown to be flexible. Bao et al. [19] used a printing approach analogous to drop-casting to create pH sensors with a sensitivity of −20.63 mV/pH on a SPE printed on bendable PET substrates using a solution of carbon nanotubes (CNTs) coated with PANI. Response times were on the order of several seconds. Although PET (used for plastic water bottles) is technically flexible, it does not warrant itself as well to wearables as textile substrates.

Instead of PANI, Dang et al. [11] created a fully screen-printable CNT-polyurethane composite that was both flexible and stretchable. A quick response time of 8 s was achieved, but the overall sensitivity of this sensor was greatly reduced, at −11 mV/pH. In comparison, the flexible screen-printed composites developed in this work show much greater sensitivities of −27.9 mV/pH and −42.6 mV/pH for the PANI-EB and PANI-ES composites, respectively, albeit with slower response times. Consequently, challenges still exist in creating wearable sensors on textiles in part due to their rough, porous and flexible nature.

While the addition of KCl into the pH buffer solutions used for testing somewhat models human sweat, measurements should next be attempted using real sweat samples with controlled pH values. Further studies on selectivity at various ion concentrations (sodium, potassium, chloride, sulphate, etc.) are still required. In terms of determining whether the PANI composite pH sensor can be used as a reusable device, testing its ability to withstand multiple wash cycles represents the next step in development. While we have rinsed each of the sensors repeatedly with de-ionized water, we have not performed formal washability studies (e.g., [22]). Reproducibility of the sensor is also an important parameter that needs to be characterized for the sensor to move beyond the prototype stage into a manufacturable device.

Additionally, implementation of a more stable RE would be required for measurements occurring over a longer time span, as some drift was observed in the sensor response. The importance of a solid electrolyte layer has been discussed elsewhere [28].

Nevertheless, this technology has the potential to be used as a real-time pH sensor for applications such as the monitoring of infected wounds, where time scales are over the course of days, so the slow response of the fully screen-printed sensors is of less concern, given its simple and cost-effective fabrication process that can be easily integrated with clothing or other textiles (e.g., bandages). The ability to create sensors using the infrastructure already available for patterning textiles, materials already worn by people, allows for more complete monitoring of susceptible patients with addressable health issues.

## 5. Conclusions

A novel, flexible, screen-printable pH-responsive PANI-DBSA composite ink has been created that can be printed on textile substrates for use in wearable devices. The composite shows promise to create a fully screen-printed pH sensor that can additionally be isolated by means of enclosed microfluidic channels printed on the textile substrate for fully screen-printed microfluidic sensor systems. The sensitivities achieved by the screen-printed PANI composites exceed that of previously reported flexible fully screen-printed sensors, approaching that of surface functionalized working electrodes, albeit at the expense of response time. Nevertheless, the simpler one-process fabrication of this technology lends itself well to the ease of manufacturing of these devices.

## Figures and Tables

**Table 1:** Summary of formulations and resulting thicknesses for PANI-EB + DBSA + Speedball composites.

PANI wt%	DBSA wt%	Speedball Ink wt%	Average Thickness (μm)	Qualitative Print Quality
5	5	90	48.3	Good
10	10	80	54.5	Good
15	15	70	67.4	Good
17.5	17.5	65	56.6	Good
20	20	60	59.8	Good
25	25	50	62.5	Good
30	30	40	57.1	Good
35	35	30	51.8	Ok—slight cracking during handling
40	40	20	79.8	Bad—cracking during handling
50	50	0	44.6	Bad—cracking, unprintable

**Table 2:** Curve-fitting, linearity, and sensitivity data corresponding to Figure 12c.

*Working Electrode*	*Regression Lines*	*R^2*	*Slope*	*Sensitivity (mV/pH)*
*SP-ES*	*y = −0.0428x + 0.4438*	*0.9511*	*−0.0428*	*−42.8*
*SP-EB*	*y = −0.0282x + 0.3448*	*0.9971*	*−0.0282*	*−28.2*
*DC*	*y = −0.0554x + 0.3838*	*0.9954*	*−0.0554*	*−55.4*
*EP*	*y = −0.0425x + 0.2665*	*0.9779*	*−0.0425*	*−42.5*

**Table 3:** Curve-fitting, linearity, and sensitivity data corresponding to Figure 16.

State	Regression Lines	R^2^	Slope	Sensitivity (mV/pH)
Initial	y = −0.0326x + 0.3453	0.9328	−0.0326	−32.6
After 50 bend cycles	y = −0.0321x + 0.3238	0.9629	−0.0321	−32.1
After 100 bend cycles	y = −0.0332x + 0.3216	0.9715	−0.0332	−33.2

**Table 4:** Comparison of fabrication, flexibility, sensitivities, and response times of sensors described herein and select literature values. It should be noted that the sensors described herein achieve 90% of their steady state OCP response in less than half the listed response times listed.

Sensor	Fabrication	Flexible	Textile Substrate	Sensitivity (mV/pH)	Response Time (s)
Screen-Printed Composite PANI-EB	Entirely screen/stencil printed	Yes	Yes	−27.9	1245
Screen-Printed Composite PANI-ES	Entirely screen/stencil printed	Yes	Yes	−42.6	2724
Electropolymerized PANI on carbon-based SPE	WE electropolymerized on SPE	Yes	Yes	−55.4	521
Drop-Casted PANI on carbon SPE	WE drop-casted on SPE	Yes	Yes	−41.3	581
Screen-Printed Graphite-Polyurethane Composite [11,28]	Screen/Stencil printed	Yes	No	−4 [28], −11.18 [11]	5
PANI-PVB-PS3 Composite [21]	Screen-printed	No	No	60 Ω/pH (conductimetric sensor)	60–120
Electropolymerized PANI on carbon-based SPE [13]	WE electropolymerized on SPE	Yes	Yes	−59.2	<20

## Data Availability

The data presented in this study are available upon request from the corresponding author.

## References

[1] Simmers P., Li S.K., Kasting G., Heikenfeld J. (2018). Prolonged and localized sweat stimulation by iontophoretic delivery of the slowly-metabolized cholinergic agent carbachol. J. Dermatol. Sci..

[2] Hatano Y., Man M.-Q., Uchida Y., Crumrine D., Scharschmidt T.C., Kim E.G., Mauro T.M., Feingold K.R., Elias P.M., Holleran W.M. (2009). Maintenance of an Acidic Stratum Corneum. J. Investig. Dermatol..

[3] Curto V.F., Fay C., Coyle S., Byrne R., O’Toole C., Barry C., Hughes S., Moyna N., Diamond D., Benito-Lopez F. (2012). Real-time sweat pH monitoring based on a wearable chemical barcode micro-fluidic platform incorporating ionic liquids. Sens. Actuators B Chem..

[4] Schneider L.A., Korber A., Grabbe S., Dissemond J. (2006). Influence of pH on wound-healing: A new perspective for wound-therapy?. Arch. Dermatol. Res..

[5] Brown M.S., Ashley B., Koh A. (2018). Wearable technology for chronic wound monitoring: Current dressings, advancements, and future prospects. Front. Bioeng. Biotechnol..

[6] Alam A.U., Qin Y., Nambiar S., Yeow J.T., Howlader M.M., Hu N.-X., Deen J. (2018). Polymers and organic materials-based pH sensors for healthcare applications. Prog. Mater. Sci..

[7] Manjakkal L., Szwagierczak D., Dahiya R. (2020). Metal oxides based electrochemical pH sensors: Current progress and future perspectives. Prog. Mater. Sci..

[8] Stuart T., Hanna J., Gutruf P. (2022). Wearable devices for continuous monitoring of biosignals: Challenges and opportunities. APL Bioeng..

[9] Bandodkar A.J., Hung V.W.S., Jia W., Valdés-Ramírez G., Windmiller J.R., Martinez A.G., Ramírez J., Chan G., Kerman K., Wang J. (2013). Tattoo-based potentiometric ion-selective sensors for epidermal pH monitoring. Analyst.

[10] Chung D., Gray B.L. (2019). Editors' Choice—Development of Screen-Printed Flexible Multi-Level Microfluidic Devices with Integrated Conductive Nanocomposite Polymer Electrodes on Textiles. J. Electrochem. Soc..

[11] Dang W., Manjakkal L., Navaraj W.T., Lorenzelli L., Vinciguerra V., Dahiya R. (2018). Stretchable wireless system for sweat pH monitoring. Biosens. Bioelectron..

[12] Rahimi R., Ochoa M., Tamayol A., Khalili S., Khademhosseini A., Ziaie B. (2017). Highly Stretchable Potentiometric pH Sensor Fabricated via Laser Carbonization and Machining of Carbon−Polyaniline Composite. ACS Appl. Mater. Interfaces.

[13] Guinovart T., Valdés-Ramírez G., Windmiller J.R., Andrade F.J., Wang J. (2014). Bandage-Based Wearable Potentiometric Sensor for Monitoring Wound pH. Electroanalysis.

[14] Yoon J.H., Hong S.B., Yun S.-O., Lee S.J., Lee T.J., Lee K.G., Choi B.G. (2017). High performance flexible pH sensor based on polyaniline nanopillar array electrode. J. Colloid Interface Sci..

[15] Park H.J., Yoon J.H., Lee K.G., Choi B.G. (2019). Potentiometric performance of flexible pH sensor based on polyaniline nanofiber arrays. Nano Converg..

[16] Cho S., Lee J.S., Joo H. (2019). Recent Developments of the Solution-Processable and Highly Conductive Polyaniline Composites for Optical and Electrochemical Applications. Polymers.

[17] Lindfors T., Ivaska A. (2002). pH sensitivity of polyaniline and its substituted derivatives. J. Electroanal. Chem..

[18] Bocchini S., Castellino M., Della Pina C., Rajan K., Falletta E., Chiolerio A. (2018). Inkjet printed doped polyaniline: Navigating through physics and chemistry for the next generation devices. Appl. Surf. Sci..

[19] Bao Q., Yang Z., Song Y., Fan M., Pan P., Liu J., Liao Z., Wei J. (2019). Printed flexible bifunctional electrochemical urea-pH sensor based on multiwalled carbon nanotube/polyaniline electronic ink. J. Mater. Sci. Mater. Electron..

[20] Sha R., Komori K., Badhulika S. (2017). Amperometric pH Sensor Based on Graphene–Polyaniline Composite. IEEE Sens. J..

[21] Gill E., Arshak A., Arshak K., Korostynska O. (2010). Response mechanism of novel polyaniline composite conductimetric pH sensors and the effects of polymer binder, surfactant and film thickness on sensor sensitivity. Eur. Polym. J..

[22] Chung D., Khosla A., Gray B.L. (2014). Screen printable flexible conductive nanocomposite polymer with applications to wearable sensors. Nanosens. Biosens. Info-Tech. Sens. Syst..

[23] Laffitte Y., Gray B.L. Real-time potentiometric pH-sensor using a screen-printable polyaniline composite on textiles. Proceedings of the 2021 IEEE International Conference on Flexible and Printable Sensors and Systems (FLEPS).

[24] Laffitte Y., Gray B.L. 3-D cloth-based microfluidic devices with embedded sensors. Proceedings of the CBMS 2021 International Conference on Miniaturized Systems for Chemistry and Life Sciences (µTAS).

[25] Aoki K.J., Chen J. (2018). Tips of voltammetry. Voltammetry.

[26] Baker L.B. (2019). Physiology of sweat gland function: The roles of sweating and sweat composition in human health. Temperature.

[27] Khosla A., Gray B.L. (2009). Preparation, characterization and micromolding of multi-walled carbon nanotube polydimethylsiloxane conducting nanocomposite polymer. Mater. Lett..

[28] Manjakkal L., Dervin S., Dahiya R. (2020). Flexible potentiometric pH sensors for wearable systems. RSC Adv..

